# Circulating exosomal miR-125a-3p as a novel biomarker for early-stage colon cancer

**DOI:** 10.1038/s41598-017-04386-1

**Published:** 2017-06-23

**Authors:** Jing Wang, Feihu Yan, Qi Zhao, Fei Zhan, Ruitao Wang, Liang Wang, Yanqiao Zhang, Xiaoyi Huang

**Affiliations:** 1 Department of Oncology, The Second Affiliate Hospital of Harbin Medical University, Harbin, 150081 China; 2 Department of Gastrointestinal Medical Oncology, Harbin Medical University Cancer Hospital, Harbin, 150081 China; 3 Department of Internal Medicine, Harbin Medical University Cancer Hospital, Harbin, 150081 China; 4 Department of Pathology and MCW Cancer Center, Medical College of Wisconsin, Milwaukee, WI 53226 USA; 5 Biotherapy Center, Harbin Medical University Cancer Hospital, Harbin, 150081 China; 6 Center of Translational Medicine, Harbin Medical University, Harbin, 150086 China

## Abstract

Circulating exosome holds great potentials as biomarker for diagnosis and prognosis of human cancers. Previously, we have applied small RNA sequencing to identify aberrantly expressed exosomal miRNAs as candidates for diagnostic markers in colon cancer patients. In this validation cohort, plasma derived exosomal miRNA was isolated from 50 early-stage colon cancer patients and 50 matched healthy volunteers. Real-time qRT-PCR revealed that miR-125a-3p, miR-320c were significantly up-regulated in plasma exosomes of the patients with early stage colon cancer. ROC curve showed that miR-125a-3p abundant level may predict colon cancer with an area of under the curve (AUC) of 68.5%, in comparison to that of CEA at 83.6%. Combination of miR-125a-3P and CEA improved the AUC to 85.5%. In addition, plasma exosome level of miR-125a-3p and miR-320c showed significant correlation with nerve infiltration (P < 0.01), but not with tumor size, infiltration depth, and differentiation degree (P > 0.05). On the contrary, plasma CEA level is correlated with tumor size, infiltration depth, and differentiation degree (P < 0.05, r = 0.3009–0.7270), but not with nerve infiltration (P = 0.744). In conclusion, this follow-up study demonstrated circulating plasma exosomal miR-125a-3p is readily accessible as diagnosis biomarker for early-stage colon cancer. When combined with conventional diagnostic markers, miR-125a-3p can improve the diagnostic power.

## Introduction

Prognosis of colorectal cancer (CRC) is dependent on the disease stages at diagnosis, with a 5-year survival rate of 90% when diagnosis early and less than 10% when distant metastases developed^1^. Diagnosis in early stage colon cancer is not common because flexible colonoscopy, the authorized method for diagnosis of colon cancer, is not available in routine physical examination. In addition, flexible colonoscopy is invasive and uncomfortable, making patients (especially female patients) feel shy or fear to have the examination through the anus. Moreover, flexible colonoscopy is difficult to be carried out on elderly with heart or blood diseases which prohibit patients from invasive examination. Serum serological tumor markers CA199 and CEA are useful for detecting colorectal cancer^2^, yet with low specificity^3–5^. Therefore, there is urgent need to develop optimal markers for rapid and highly sensitive screening of colon cancer^6^. With an increasing availability of therapeutic options for colon cancer, knowledge of predictive biomarkers has also become clinically relevant to identify early-stage patients destined to benefit more from therapeutic intervention. This potentially allows for tailoring specific treatments for responsive patient groups, and spares the morbidity of adverse effects from treatment to patient groups who are unlikely to benefit from the specific colon cancer stage treatment. Cell free nucleic acid profiling in circulatory fluids offers a potential for predictive biomarker development in various cancers^7^.

Circulating exosomes in the blood are small (30–100 nm) membrane vesicles released into the extracellular environment on fusion of multivesicular bodies with the plasma membrane^8^. It is reported that exosomes contain proteins and RNAs (in particular, small non-coding RNAs) selectively enriched from parent cells^9^. The exosomal outer membrane protects RNA contents from being degraded while circulating throughout the body, offering the opportunity to be developed as cancer prognostic and predictive biomarkers. A number of exosomal RNAs in the plasma are being developed as diagnostic, prognostic, or even therapeutic biomarkers in patients with various tumor types such as ovarian cancer^10^, prostate cancer^11, 12^, as well as colon cancer^13^. Exosomal miR-1229, miR-1246, miR-150, miR-21, miR-223, and miR-2a are reported to be significantly up-regulated in CRC patients^13^. Previously by applying RNA sequencing to discover and characterize profile of plasma-derived exosomal miRNAs of CRC, we have identified a series of diagnostic candidates^14, 15^. These studies clearly demonstrated that circulating miRNAs, especially exosome-enclosed miRNAs, may aid in diagnostic of CRC. However, clinical application of exosomal RNAs in early-stages of colon cancer is still uncertain and many new miRNA markers are needed to be verified.

In this follow up study, we selected a total of 10 plasma exosomal miRNA markers determined by RNA sequencing in early-stage colon cancer and validated these markers in newly recruited 50 early-stage CRC patients and matched healthy controls. We evaluated the diagnostic power of these markers in comparison to the clinical and pathological characteristics of these patients. The result of this study showed that some circulating miRNAs may complement existing predictor to improve diagnosis of early-stage colon cancer.

## Results

### Characteristics of exosomes and exosomal miRNA in plasma

**Figure 1:**
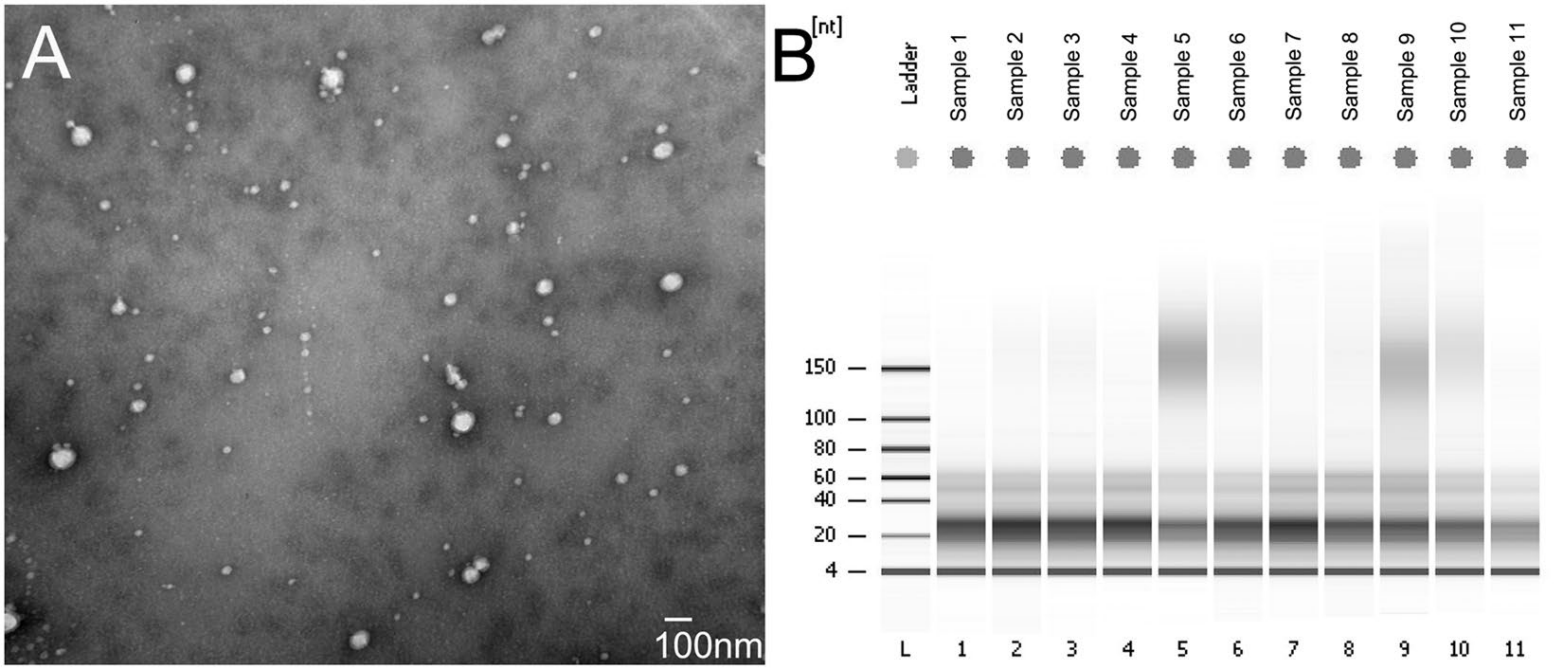
Plasma exosomes and their RNA sizes. (**A**) Scanning electron microscopy revealed the phenotype of the exosomes isolated from plasma. Exosomes isolated with ExoQuick were fixed and processed with STM. The bar represents 100 nm. (**B**) Exosomal RNAs determined by small RNA chip. A total of 50 μL exosome solution from 250 μL plasma was subjected to RNA extraction. The column binding RNA was resolved in 14 nuclease free water. One microliter of the RNA solution was added in the small RNA chip and processed with Bioanalyzer 2100. Shown is the representative gel electrophoresis map.

To quantify the exosomes isolated with the ExoQuick Exosome Precipitation Solution, we examined exosome using transmission electron microscopy to determine the size distribution. As shown in Fig. 1A, the sizes of the photo-captured round-shaped microvesicles were mainly in about 100 nm in diameter in the exosome-enriched fraction. Next, exosomal RNA was purified and RNA quantity and quality were determined by the Bioanalyzer using Small RNA Chip (Fig. 1B). With 250 μL plasma, miRNA yields were from 8 to12 ng with median 10.4 ng, slightly lower than our previous isolation under same condition^14^. Also from Fig. 1B, miRNA within length of 18–28 nt, peak at 22 nt, were the dominant small RNA species in the exosomes.

### MiR-125-3p and miRNA-320c can distinguish early-stage colon cancer from the healthy controls

**Figure 2:**
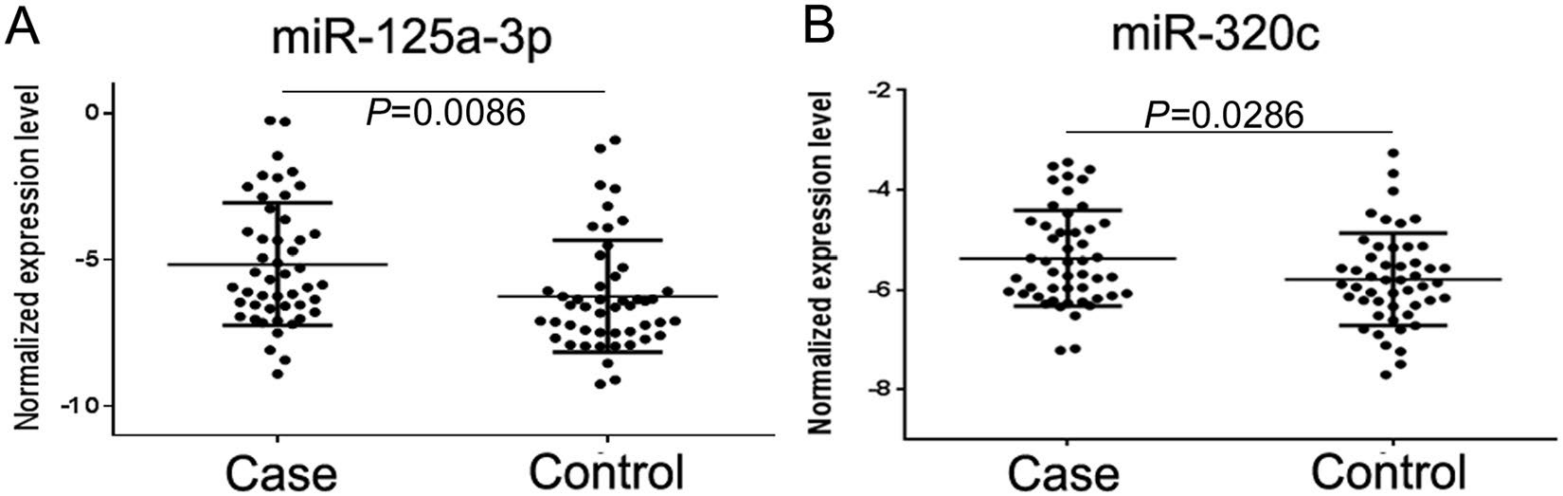
Expression levels of the miRNA candidates in the CRC patients and the healthy controls. A total of 3ng miRNA was reverse transcribed using QIAGEN miScript II RT kit. After 1:10 dilution, two microliters of cDNA template were used for quantification by real-time PCR. MiR-30e-5p was used as internal control. Relative expression levels of miR-125a-3p (**A**) and miR-320c (**B**) in the CRC patients (Case) and healthy controls (Control) are shown.

Based on the data from RNA sequencing, the candidate miRNA biomarkers are listed in Table 1 ^15^. In this follow up study, we selected nine miRNA candidates (miR-125-3p, miR-320c, miR-320d, miR-9-1, miR-139, miR-125a-5p, miR-4792, miR-376, miR-543, miRNA-381) for validation in the independently recruited patients with early-stage (I, II) colon cancer. The clinical characteristics of the 50 patients are shown in Table 2. Age and sex matched healthy individuals were used as controls. Amplification of miR-30e-5p, which was universally expressed between the two groups (Figure S1), was served as internal normalization standard. Among the selected miRNAs, miR-125a-5p was barely detectable, consistent with its relative low copy in the RNA sequencing dataset. Detection of miR-4792 and miR-139 significantly varied across technical triplicates possibly due to its high Ct value beyond 35. Levels of miR-376, miR-543, and miRNA-381 were undetectable in most of the samples with QIAGEN miScript Primer Assay. MiR-9-1, miR-320d, miR-125-3p, and miR-320c were the only four miRNAs that could be stably amplified. After normalization to miR-30e-5p, examination of the relationship between the early stage colon cancer and each marker demonstrated a statistically significant up-regulation of miR-125-3p (Fig. 2A, *P* = 0.018) and miR-320c (Fig. 2B, P = 0.028) in colon cancer patients when compared to healthy controls. However, similar statistical trend was not observed between MiR-9-1, miR-320d and cancer subtype.

**Table 1:** Early stage colon cancer relevant miRNA markers (Stage I and Stage II compared with normal controls).

Column ID	p-value	Mean (CC)	Mean (N)	MeanRatio (CC/N)	Description
miR-1343	0.001229	6.36552	6.597	0.851758	Down
miR-125a-5p	0.002274	10.5739	10.8508	0.825371	Down
miR-376a-3p	0.002799	4.32724	4.56082	0.850522	Down
miR-381-3p	0.003413	12.8012	12.9714	0.888673	Down
miR-543	0.003669	8.78779	9.00972	0.857414	Down
miR-128	0.004384	15.7208	15.9592	0.847689	Down
miR-139-5p	0.0045	9.85424	10.0315	0.884386	Down
miR-9-5p	0.039994	14.2345	14.4432	0.865361	Down
miR-125a-3p	0.003511	9.13797	8.99687	1.10275	Up
miR-320c	0.007102	8.53069	8.28988	1.18166	Up
miR-320d	0.0084	6.94879	6.70643	1.18292	Up
miR-4792	0.0088	5.25523	5.02512	1.17293	Up
miR-320b	0.009362	10.8133	10.584	1.17228	Up
miR-320a	0.009647	15.325	15.0996	1.16913	Up
miR-378d	0.024233	8.18391	8.01852	1.12146	Up
miR-378a-3p	0.028654	13.6745	13.5519	1.08872	Up
miR-1246	0.030753	6.43356	6.16737	1.20263	Up
miR-378c	0.03253	8.58289	8.42201	1.11797	Up

CC = Colon Cancer; N = Normal Control.

**Table 2:** Clinical characteristics of early-stage colon cancer patients.

	Validation Cohort
Total number	50
**Gender**	
Female	18
Male	32
**Age at enrollment**	
Median	62.3
Range	48–75
**tuomor location**	
right hemicolon	18
left hemicolon	32
**Stage at enrollment**	
stage I	3
stage II A	43
stage II B	4
**TNM Staging**	
T1	0
T2	4
T3	42
T4	4
Tx	0
**N**	
N0	50
N1	0
N2	0
N3	0
Nx	0
**M**	
M0	0
M1	0
**nerve infiltrate**	
+	7
−	39
unknown	4

### MiR-125-3p enhances the diagnostic power of CEA for early stage colon cancer

**Figure 3:**
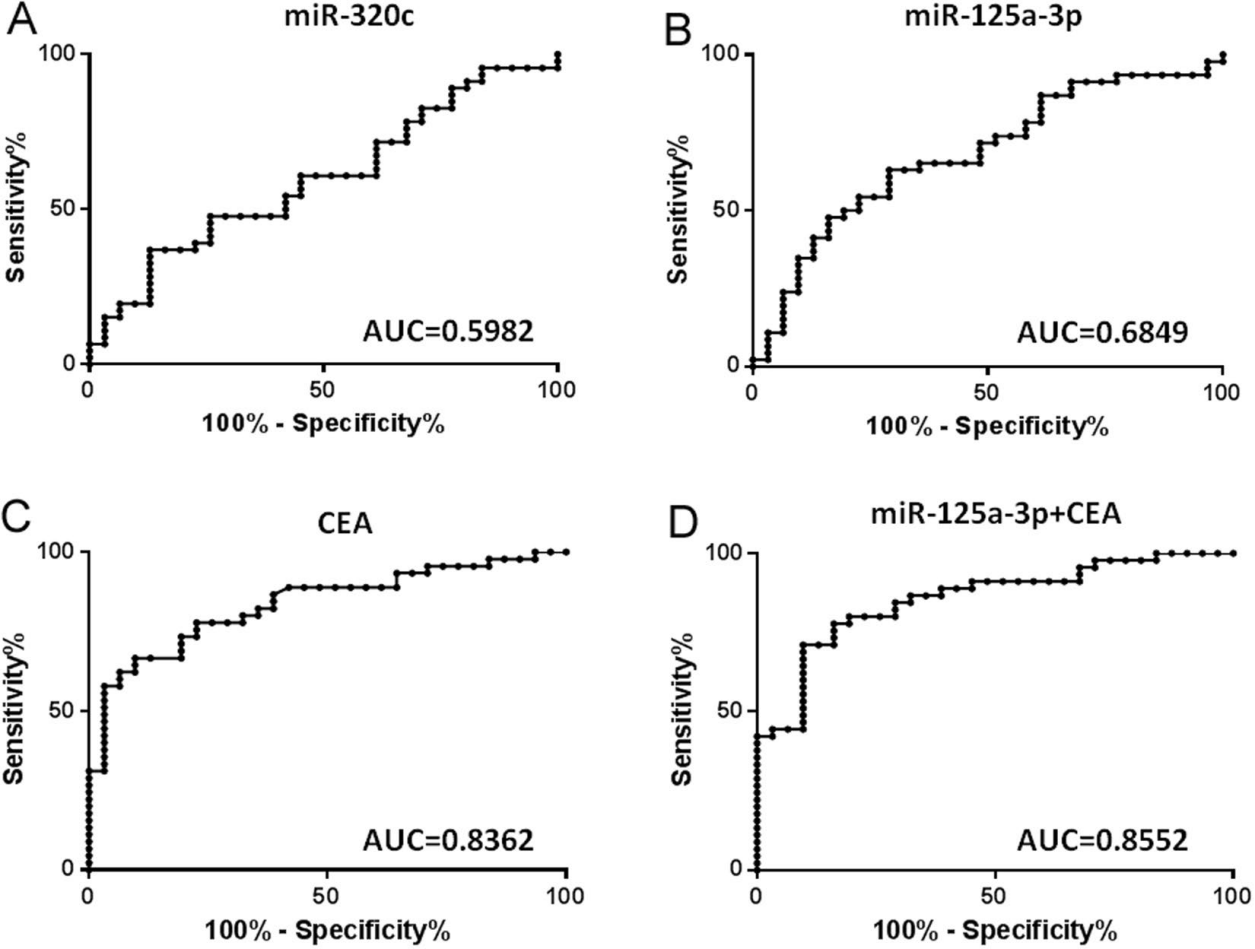
Diagnostic power of each tumor marker determined by ROC curve. Serum level of CEA and the expression level of miR-320c and miR-125a-5p were respectively stratified into high and low group with their geometric mean as cut-off. The performance of miR-30c (**A**), miR-125a-3p (**B**), CEA (**C**), and miR-125a-3p plus CEA (**D**) yielded area under the ROC curve (AUC) values of 0.5982 (*P* < 0.1459), 0.6849 (*P* = 0.0156), 0.8362 (*P* < 0.0001), and 0.8552 (*P* < 0.0001).

To determine the diagnostic power of the exosome miRNAs and conventional tumor marker CEA for early-stage colon cancer, we examined miRNA expression levels for their capacity of distinguishing patients from healthy controls. As shown in Table 3 and Fig. 3, the performance of miR-125a-3p yielded area under the ROC curve (AUC) values of 0.6849 (Fig. 3A, *P* < 0.001) and miR-320c with AUC = 0.5982 (Fig. 3B, P = 0.1459), in comparison to CEA with AUC = 0.8362 (Fig. 3C, *P* < 0.0001). Significant synergistic effect was found when the up regulation of miR-125a-3p was combined with CEA level, which generated an AUC of 0.8552 (Fig. 3D, *P* < 0.0001). Our results suggest that the best predictive model was the combination of miR-125a-3p and CEA.

**Table 3:** Diagnostic power of each marker for early-stage colon cancer.

	AUC	P	95%CI
miR-125a-3p	0.6849	0.0074	0.5593–0.8025
miR-320c	0.5982	0.1459	0.4705–0.7259
CEA	0.8362	<0.0001	0.7467–0.9257
miR-125a-3p + CEA	0.8552	<0.0001	0.7707–0.9397

AUC = Area Under the ROC curve.

CI = Confidence Interval.

### Correlations between exosomal miRNAs and clinical pathology characteristic

**Figure 4:**
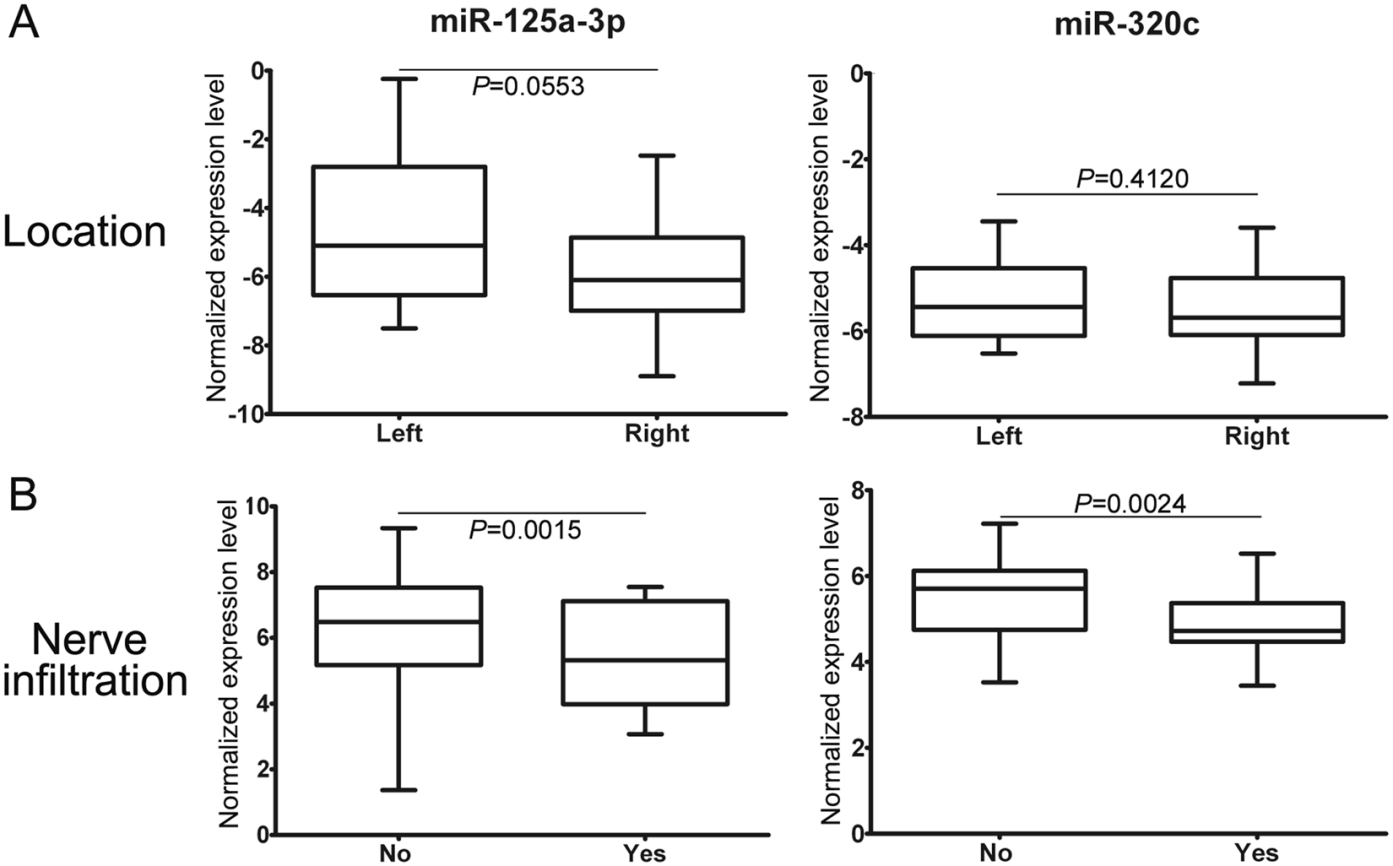
Relationship between the tumor markers and the clinical characteristics. Shown are statistically analysis on trend between miR-125a-3p versus tumor location and nerve infiltration (**A**), and miR-320c versus tumor location and nerve infiltration (**B**). Boxes show the range from first to third quartiles and are divided by the line of median (second quartile). The plus and minus whiskers indicate the maximum and minimum, respectively.

We further examined the relationship between the miRNA markers and the clinical pathology characteristics of colon cancer patients. Correlation analysis showed that miR-125a-3p and miR-320c were not correlated with tumor size, infiltration depth, and differentiation status (Tables 4 and S1). As shown in Table 4 and Fig. 4A, miR-125a-3p demonstrated a borderline statistical significance to be up regulated in the left colon cancer when compared to the right (*P* = 0.0553). However, miR-320c didn’t show any difference between left and right colon cancer (*P* = 0.4120). Interestingly, both miR-125a-3p (*P* = 0.0015) and miR-320c (*P* = 0.0024) were significantly lower in colon cancer patients with nerve infiltration when compared to those without nerve infiltration (Table 4 and Fig. 4B).

**Table 4:** Correlation between the clinical parameters and the markers.

	p-value	t	Mean	Mean	MeanRatio	Mean Diff	Fold Change
**differentiation (Moderate vs. Poor)**							
miR-125a-3p	0.6903	−0.4011	−5.2258^a^	−4.8505^b^	0.7710	−0.3753	−1.2971
miR-320c	0.5967	0.5330	−5.3410^a^	−5.5672^b^	1.1697	0.2261	1.1697
CEA	0.0036	−3.0761	5.1752^a^	72.5380^b^	0.0713	−67.3628	−14.0250
**nerve infiltration (No vs. Yes)**							
miR-125a-3p	0.0015	−3.4026	−5.5010^c^	−2.8089^d^	0.1547	−2.6920	−6.4623
miR-320c	0.0024	−3.2199	−5.5259^c^	−4.3550^d^	0.4442	−1.1708	−2.2514
CEA	0.7440	0.3286	13.5773^c^	6.1600^d^	2.2040	7.4173	2.2040
**localization (Left vs. Right)**							
miR-125a-3p	0.0553	1.9960	−4.7260^e^	−5.9320^f^	2.3069	1.2060	2.3069
miR-320c	0.4120	0.8278	−5.2890^e^	−5.5280^f^	1.1794	0.2381	1.1794
CEA	0.3330	0.9982	5.2580^e^	25.2700^f^	0.2081	−20.0100	−4.8050

^a^Mean of moderate, ^b^mean of poor, ^c^mean of without infiltration, ^d^mean of with infiltration, ^e^mean of left colon cancer, ^f^mean of right colon cancer.

## Discussion

Despite advances in treatment for colorectal malignancies, clinicians are still facing the challenge of how to improve diagnosis at the early or pre-early stages of the disease by developing methods that can detect malignancies, while bypassing the side effects of biopsy and other conventional diagnostic approaches. It is also imperative that active, accurate, and attainable surveillance to optimize the clinical outcome should be enforced during the whole treatment process. In addition, early detection of cancer is the critical factor for successful treatment and reduction of mortality. In these regards, evidenced by substantial literature references in many cancers^16–20^, extracellular miRNAs embedded in microvesicles such as circulating exosomes are capable of non-invasively providing diagnostic, prognostic, or even therapeutic targets for CRC.

In this study, we selected miR-9-1, miR-125a-3p, miR-125a-5p, miR-320c, miR-4792, miR-376, miR-543, and miR-381 as diagnostic candidates for validation. Among these candidates, miR-125a-3p and miR-320c were the only two miRNAs that could be stably amplified using the commercial available primer sets from QIAGEN. Based on our experience, the quantification assays play determinant role in the detectability of a unique miRNA enclosed in plasma exosome. For example, real-time amplification of miR-375-5p could be steadily achieved using QIAGEN miScript Primer Assay, while always failed using TaqMan MicroRNA Assays from Life technologies^11^. However, miR-141 showed poor amplification using same reaction system from QIAGEN, no matter how the optimized approaches were invested. Different detection mechanisms between assays may contribute to the amplification bias. In addition, miRNAs also show sequence length variations with respect to the reference sequences. This variation, called isomiR, may create significant problems for TaqMan-based miRNA assay. Relatively low copy number of unique miRNA may be another reason for poor detectability. According to our experience, low abundant miRNA with average normalized read counts of <32, which is equivalent to 2^5^ among the readout of sequencing data set, should be precluded from real-time PCR in order for the ease of validation. Taken together, it’s not likely to guarantee all of the miRNA markers determined with NGS in the discovery stage could be successfully detected using real-time PCR approaches in the subsequent validation stage. Relatively larger number of miRNA candidates should be seriously considered when a mining of miRNA biomarker moves onto the point of technical transition^21, 22^. In addition, more exosomal miRNA candidates are needed to find additional biomarkers with potential for screening of early stage CRC, because the diagnostic potency of multiple molecules is much stronger than single molecule^23–26^.

For miRNA quantification, internal controls to normalize expression level has been questionable^27^. Especially for the exosomal miRNA, the widely accepted relative control RNU6B is absent from detection even using RNA sequencing platform^11^. To screen for best endogenous miRNA controls, we analyzed our exosomal RNA sequencing data and found miR-30e-5p, and miR-30a-5p were among the top 10 most stably expressed miRNAs with the least inter-group and intra-group variations^11^. In this study, absolute expression level of circulating exosomal miR-30e-5p in our newly recruited samples showed no statistical differences between the case group and the healthy control group, further supporting the eligibility of miR-30e-5p as an endogenous standard for miRNA quantification. To date, performance of miR-30e-5p for relative normalization has been tested in more than 400 individuals and will be continuously assessed in larger sample size and in multiple research centers.

Using miR-30e-5p as endogenous normalizer, we found that level of exosomal miR-125a-3p significantly elevated in patients with early stage CRC (p = 0.0074). miR-125 family, a highly conserved miRNA family throughout evolution, is consist of miRNA-125a-3p, miR-125a-5p, miR-125b-1 and miR-125b-2. The aberrant expression of miR-125 family is tightly related to tumorigenesis and tumor development[13]. Aberrant expression of miR-125a-3p has been documented in serum of patients with pancreatic cancer, biliary-tract cancer, systemic lupus erythematosus, and moyamoya disease^28–30^. MiR-125a-3p expression reduced migration and increased apoptosis of prostate cancer cells by targeting Fyn, FAK and paxillin^31, 32^. In lung cancer, miR-125a-3p induces apoptosis of cancer cell via p53 dependent and p53 independent ways^33^. A study showed that PI3K/AKT/mTOR pathway is involved in miR-125a-3p induced reduction of the migration and invasion of liver cancer^34^. In addition to direct action on tumorigenesis, miR-125a-3p was found to increase chemosensitivity of breast cancer cell by 3′-UTR of *BRCA1* ^35^. These evidences collectively indicated miR-125a-3p may play its multiple roles as tumor inhibitor. Functional role of miR-125a-3p in the process of CRC remains to be fully elucidated.

In general, the diagnostic accuracy of combined markers will be higher than single one^25^. For early diagnosis of CRC, A panel of four-miRNA signature consisting of miR-23a-3p, miR-27a-3p, miR-142-5p and miR-376c-3p was established with AUC of 0.917^23^. Another study demonstrated that the serum exosomal levels of seven miRNAs (let-7a, miR-1229, miR-1246, miR-150, miR-21, miR-223, and miR-23a) were significantly higher in CRC patients^13^. By combining with CEA and miR-125a-3p, our multivariant model shows an increased diagnostic power with AUC = 0.855. The above evidence, together with ours, firmly suggests the rationale that circulating exosomal miRNA as predictor for CRC and addition of these miRNAs to current testing regimens may improve diagnosis accuracies. Given optimized, accessing the level of a single miRNA marker, combined with or without other miRNA or conventional tumor marker, will be a convenient, efficient, and cost-effective screening method for healthy population.

Clinical research has shown that left and right colon cancers react differently to the same chemotherapy scheme^36, 37^. We also analyzed the correlation between the level of the selected miRNA candidates and the location of lesion. We showed that there was a trend that miR-125a-3p is higher in patient with left CRC than those with right CRC. This result suggests that molecular signature of the right CRC may different from that of the left. In addition to the location, we also found significant correlation of up-regulation of miR-125a-3p with non-tumor infiltration, whereas CEA is associated with tumor size, infiltration depth, and differentiation degree, which is consistent with results from recent study^2^. These results indicate that expression of miR-125a-3p is independent from CEA and miR-125a-3p may play critical roles when CRC invades the peripheral nerves.

## Conclusion

Our follow-up study confirmed one miRNA candidate (miR-125a-3p) with potential for screening patients with early-stage CRC when combining with other diagnostic test. This plasma-based non-invasive approach may readily complement current conventional invasive detection approaches. With further optimization, the plasma exosomal miR-125a-3p may provide additional option to distinguish the early-stage CRC from healthy controls.

## Materials and Methods

### Patients and samples

The inclusion criteria were as follows: All patients were newly diagnosed as early stage colon cancer (stage I, II), which was confirmed by a pathologist after operation. Patients with chemotherapy or radiotherapy before blood collection were excluded. Tumor was staged according to the International Union against Cancer (UICC) guidelines. The Clinical Research Ethics Committee of the Third Affiliated Hospital of Harbin Medical University approved the research protocols (CREC). Written informed consents were obtained from the participants before sampling. All methods were performed in accordance with the guidelines and regulations of the CREC.

In this validation cohort, plasma samples from 50 additional colon cancer patients and 50 age and sex matched healthy individuals were tested for candidate miRNAs selected from the discovery cohort^15^. Clinic characteristics of the cancer patients enrolled in this study were summarized in Table 1. Blood samples from CRC patients and controls were collected in 10 ml PST plasma separator tubes and processed within 30 minutes after collection. All specimens were centrifuged at 3,000 g for 10 minutes to generate plasma. The plasma was then fractioned into multiple aliquots and stored at −80 °C with no aliquots undergoing freeze-thaw cycles prior to exosome and RNA isolation.

### Exosome isolation

Exosomes were isolated using the ExoQuick Exosome Precipitation Solution (SBI, Mountain View, CA) as previously described^14^. Briefly, the plasma was thawed on ice and centrifuged at 3000 g for 10 min to remove possible residual cell debris. After incubated with thromboplastin D (Thermo, Middletown, CA) at 37 °C for 15 min and centrifuged, the 250 μL supernatant was aspirated to a new tube and mixed with 65 μL ExoQuick Solution. To digest free RNAs outside of exosomes, RNaseA (Sigma, St. Louis, MO) was added to the mixture at final concentration of 10 µg/ml. After keeping the mixture at 4 °C overnight, Murine RNase inhibitor (NEB, Ipswich, MA) was added at 150 units/ml before the precipitation of exosomes by centrifuging at 1500 g for 30 min. The exosome pellet was dissolved in 50 µL PBS and subjected to RNA extraction immediately.

### Scanning electron microscopy

Exosomes were fixed in 2% paraformaldehyde solution and then diluted with distilled water in serial dilutions. Five microliters of the diluted mixtures were transferred to a cleaned silicon chips. After sonication, the chips were sequentially immersed in ethanol and distilled water for 5 min in each solvent. The microvesicles were immobilized by blown dry under a ventilation hood. Then the silicon chips were mounted on a SEM stage with carbon paste. To make surface conductive, a coating of 2–5 nm gold-palladium alloy was applied by sputtering (SPI-Module Sputter, Eden Instruments, France) before imaging with scanning electron microscopy (S-4700, Hitachi, Japan). SEM was performed under low beam energies (5.0–10.0 kV). For best vesicle morphology under SEM, fresh isolated exosomes were fixed, immobilized, and imaged right after isolation. The photographs were processed using ImageJ software.

### RNA isolation

RNA was isolated with the miRNeasy Micro Kit (QIAGEN, Valencia, CA) following the standard protocol from the manufacturer. The extracted RNA was eluted with 14 µL of DNase and RNase-free water. RNA quantity and quality were determined by the Agilent Bioanalyzer 2100 using Small RNA Chip (Agilent Technologies, Santa Clara, CA) according to the manufacture’s instruction.

### Quantitative real-time PCR

MiR-9-1, miR-125a-3p, miR-125a-5p, miR-320c, miR-320d, miR-4792, miR-376, miR-139, miR-543, and miR-381(MS00010752, MS00008554, MS00003423, MS00041867, MS00031710, MS00045087, MS00007392, MS00003493, MS00010080, MS00004116, QIAGEN, Valencia, CA) were selected for downstream validation. miR-30e-5p(MS00009401) served as endogenous normalizer. Three nanograms of the isolated exosomal RNA were reversely transcribed using a QIAGEN miScript II RT kit with HighFlex buffer and universal RT primer. Three microliters diluted cDNA product (1:10 in H2O) were used for 10 μL real-time PCR reactions containing 5 μL QIAGEN SYBR green Master Mix, 1 μL universal downstream primer and 1 μL miRNA specific upstream primer. PCR reaction was performed on an ABI ViiA 7 platform.

### Statistic analysis

Relative miRNA expressions were calculated using the ΔΔCT method (ΔCT = CT_miR_ − CT_reference_). For the multivariate prediction model, we calculated the risk score by a linear combination of the Ct values from real-time PCR for the selected miRNAs and/or clinical variables. We applied the area under the curve (AUC) to assess the diagnostic power of the predictors. AUC can be used as an accurate measurement of the diagnostic marker; the larger the AUC, the better the prediction model. AUC = 0.5 indicates no predictive power, whereas AUC = 1 represents perfect predictive performance.

## Electronic supplementary material

Supplementary document
