# Supplementary material for: Circulating exosomal miR-125a-3p as a novel biomarker for early-stage colon cancer
Source: Sci Rep. 2017 Jun 23;7:4150. doi: 10.1038/s41598-017-04386-1 (PMC5482839; doi:10.1038/s41598-017-04386-1)
Supplement: Supplementary file 1 — Supplementary document [file 41598_2017_4386_MOESM1_ESM.pdf]

Circulating exosomal miR-125a-3p as a novel biomarker for early-stage colon cancer

Jing Wang, Feihu Yan, Qi Zhao, Fei Zhan, Ruitao Wang, Liang Wang, Yanqiao Zhang, Xiaoyi Huang

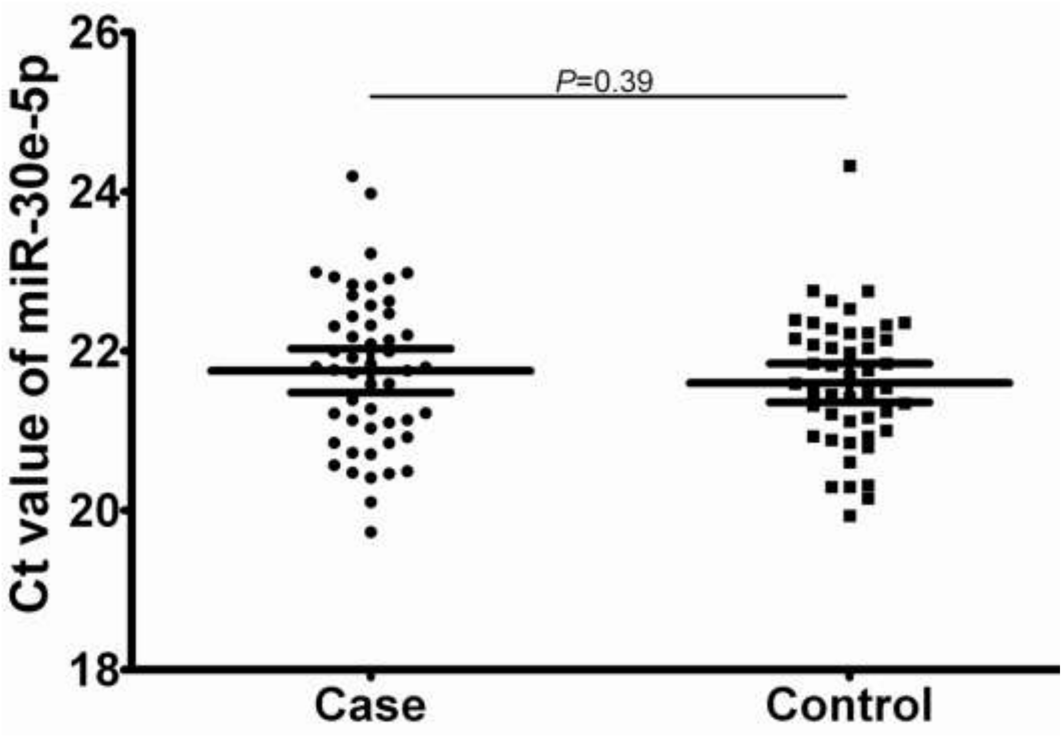

Figure S1. Distribution of miR-30e-5p abundance level across the plasma exosomes derived from CRC patients and healthy controls. Amplification of miR-30e-5p using templates isolated from plasma exosomes are described in Materials and Methods. The amount of input miRNA was 3ng. Ct values of miR-30e-5p were plotted to the CRC patients and the controls. Boxes show the range from first to third quartiles and are divided by the line of median (second quartile). The plus and minus whiskers indicate the maximum and minimum, respectively.

Table S1 Correlation between the clinical parameters and the markers-2

	p-value	r(correlation)	Lower CI	Upper CI
Correlation with tumor volume(cm^3)P				
miR-125a-3p	0.2283	-0.1899	-0.4669	0.1210
miR-320c	0.4192	-0.1264	-0.4112	0.1808
CEA	**0.0244**	0.3470	0.0481	0.5888
Correlation with infiltrate depth(cm)				
miR-125a-3p	0.3147	0.1516	-0.1451	0.4232
miR-320c	0.1817	0.1982	-0.0944	0.4592
CEA	**0.0421**	0.3009	0.0116	0.5437

CI=Confidence Interval
